# Early Childhood Education and Adult Depression: An Attrition Reanalysis With Inverse Propensity Score Weighting

**DOI:** 10.1177/0193841X20976527

**Published:** 2020-12-14

**Authors:** Christina F. Mondi, Arthur J. Reynolds, Brandt A. Richardson

**Affiliations:** 1 Brazelton Touchpoints Center, Boston Children’s Hospital, MA, USA; 2 Harvard Medical School, Cambridge, MA, USA; 3 Institute of Child Development, 5635 University of Minnesota, Minneapolis, MN, USA; 4 Human Capital Research Collaborative, 5635 University of Minnesota, Minneapolis, MN, USA; 5 St. Olaf College, Northfield, MN, USA

**Keywords:** early childhood education, preschool, depression, methodological development, measurement, inverse probability weighting, design and evaluation of programs and policies, behavioral health care and policy

## Abstract

In a previous study of the Child-Parent Centers (CPC) education program, preschool participation was linked to a 4.6 percentage point reduction (26%) in depressive symptoms at ages 22–24 over the matched comparison group enrolling the usual programs. The present study reanalyzed these data in the Chicago Longitudinal Study to address potential attrition bias since more than a quarter of the sample was missing on the outcome. Using inverse probability weighting (IPW) involving 32 predictors of sample retention, findings for the 1,142 participants growing up in high-poverty neighborhoods indicated that CPC participation was associated with a 7.1 percentage point reduction (95% CI = [−9.7, −5.4]) in one or more depressive symptoms (39% reduction over the comparison group). Although this marginal effect was within the confidence interval of the original study (95% CI = [−9.5, 0.3]), the 54% increase in the point estimate is substantial and of practical significance, suggesting underestimation in the prior study. Alternative analysis of different predictors and IPW models, including adjustments for program selection and attrition together, yielded similar results. Findings indicate that high-quality early childhood programs continue to be an important strategy for the prevention of depression and its debilitating effects on individuals and families.

Depression is a serious illness that is associated with poor social and occupational functioning and increased risk of suicide. Recent epidemiological surveys have indicated that approximately one quarter of American adults are currently experiencing depressive symptoms and that 40% or more may experience depression in their lifetime (Kessler et al., 2005; Wittayanukorn et al., 2014). The annual economic burden of depression in the United States increased by 21.5% between 2005 and 2010, for an estimated burden of USD$210.5 billion per year in medical, workplace, and suicide-related costs (Greenberg et al., 2014). The World Health Organization (2017) has also identified depression as the leading cause of disability worldwide. These findings underscore the importance of accurately quantifying the prevalence of depression and of investing in preventive interventions to reduce its toll on individuals and society.

## Early Childhood Education (ECE) Programs

ECE programs such as Head Start and the Child-Parent Centers (CPC) Preschool to 3rd Grade program provide comprehensive educational and family support services to millions of low-income children in the United States. These programs may reduce participants’ long-term risk of depression by reducing risk exposure and by promoting cumulative social, cognitive, and motivational advantage (Reynolds & Ou, 2011). Indeed, several evaluations have linked participation in high-quality ECE programs to reduced rates of depressive symptoms in emerging adulthood (e.g., McLaughlin et al., 2007; Palfrey et al., 2005; Reynolds & Ou, 2011). Reductions in depressive symptoms have also been factored into cost-benefit analyses, with findings linking ECE program participation to significant financial returns on investment (Heckman, 2011; Reynolds, Temple, White et al., 2011).

Despite these promising results, studies seeking to quantify the effects of ECE intervention on long-term depression have been limited by numerous methodological challenges. One major challenge has been overreliance on small, homogeneous study samples, which raises concerns of insufficient power to detect significant results. Another perennial challenge for longitudinal studies is *attrition* or *nonresponse*—the phenomenon in which members of an original study sample are lost in subsequent follow-ups for any reason (Carkin & Tracy, 2015). In shorter term studies, attrition commonly occurs when participants refuse to engage in follow-ups (e.g., due to concerns about the nature of the study, confidentiality, or insufficient compensation for study participation; Barry, 2005; Carkin, & Tracy, 2015). However, as study time lines grow longer (as in the case of multidecade longitudinal studies), attrition may also occur because researchers cannot get in touch with participants (e.g., due to participant residential moves or death; Barry, 2005; Carkin & Tracy).

In the realm of ECE research, several evaluation studies have maintained contact with participants for decades. For example, researchers affiliated with the Abecedarian Study (which began when participants were infants) interviewed 104 of 105 original participants who were living and eligible for inclusion at age 21. However, for ECE evaluations that work with larger samples across multiple decades, attrition is inevitable. Attrition is particularly concerning when it is *systematic*—in other words, when participants who are lost to attrition “have unique characteristics, such that the remaining sample cases cease to be representative of the original sample” (Barry, 2005, p. 267). To this end, previous research has demonstrated that participants with certain demographic characteristics are more likely to be lost to attrition over time (Härkänen et al., 2014). For example, Fitzgerald et al. (1998) analyzed data from a socioeconomically representative panel study of approximately 5,000 families and compared the characteristics of study survivors (at ages 20–38 years) to a similar sample drawn from a population survey. They reported that participants lost to attrition were more likely to be male, low-income, and to have unstable earning histories. In this way, attrition could systematically bias study results.

In order to minimize systematic attrition bias, it is important that researchers make concerted efforts to maintain contact and rapport with participants between study waves in order to minimize attrition. This requires significant time and financial resources, especially in the context of longitudinal studies that span multiple decades. When attrition cannot be prevented, advanced statistical techniques can be applied to partially correct for attrition bias.

## 2007 Study of the CPC and Adult Depressive Symptoms

The Chicago Longitudinal Study (CLS) is one of the longest running evaluations of an ECE program—in this case, the CPC program. Since 1986, CLS has followed the development of a cohort of 1,539 same-age individuals who attended Chicago Public Schools—including both CPC participants and comparison group members. Over the years, the CLS has demonstrated positive effects of CPC participation on school achievement and progress, family socialization, educational attainment, child maltreatment, and juvenile arrest (Ou & Reynolds, 2006; Reynolds, 2000; Reynolds & Robertson, 2003; Reynolds et al., 2001). Building on these findings, Reynolds and colleagues (2007) examined for the first time the relation between CPC participation and many indicators of health and well-being in early adulthood, including mental health. Probit regression analysis of a dichotomously derived self-report measure of depressive symptoms indicated that, at age 24 (*N* = 1,134), CPC preschool participants had a 4.6 percentage point lower rate of experiencing symptoms than the no-program comparison group. This difference was significant at the .057 probability level and represented a 26% reduction over the comparison group (17.4% to 12.8%) after adjusting for baseline differences in family demographic risk factors, race/ethnicity, sex, child welfare involvement, and later CPC intervention. The unique and practical significance of this estimated effect, which was consistent across a range of analyses and was not found for CPC school-age or extended program participation, led the investigators to interpret findings as an important next step in the prevention of mental health problems among economically disadvantaged youth. As the first study to link participation in a large-scale, routinely implemented preschool program with reduced rates of depressive symptoms in adulthood, these findings have direct implications for the expansion of high-quality ECE programs.

Several years later, subsequent CLS analyses expanded upon the contributions of Reynolds and colleagues’ (2007) study. A follow-up study revealed a slightly stronger estimated effect of the preschool program on depressive symptoms (12.8% vs. 17.7%; *p* = 0.04; 28% reduction) after including a propensity score for attrition as a predictor in a logit regression model (Reynolds & Ou, 2009, 2011). A structural equation modeling analysis using the five-hypothesis model of intervention found that 79% of the main effect of CPC preschool on adult depressive symptoms was accounted for by model variables, primarily through cognitive advantage, school support, and family support pathways. Reduction in depressive symptoms also made a small contribution to cost-benefit analyses of the CPC program at age 26 (Reynolds et al., 2011a).

### Addressing attrition in the CLS

As much as the findings of the Reynolds et al. (2007) study have been utilized, three limitations reduce the strength of inferences and generalizability. First, contemporary methods to correct for potential attrition bias or sample selection (e.g., inverse probability weighting, IPW) were not applied. This issue is discussed in depth below in relation to emerging IPW methods.

The second limitation of the Reynolds et al. (2007) study is that while the practical significance of the program effect was emphasized (as would be expected for such a major social problem as depression), that findings’ borderline statistical significance raises the question of the stability of effects at conventional levels. Confidence about impacts and the consequences of random fluctuations would be greater if the likelihood of chance findings is below .05. Whether the estimate is replicable, robust, and generalizable under a range of conditions warrants further analysis.

Finally, while the study did explore potential attrition bias and other model specifications in sensitivity analyses, documentation of the attrition process was not a focus of the study and was thus limited. The predictors of attrition for depressive symptoms were primarily family demographic and school performance indicators rather than a comprehensive set of factors (Mondi et al., 2017) and plausible interaction terms. Measurement error is often higher for psychological well-being than for other outcomes. This may lead to conservative bias, especially given that depressive symptoms may directly impact the likelihood of completing the adult survey. For example, participants experiencing symptoms during data collection may have been more socially isolated and economically disadvantaged (making it more difficult to locate them) or less motivated to participate. To this end, weighting the regression model accordingly may significantly reduce any potential bias stemming from differential attrition.

#### Applying IPW to the CLS

Although the CLS has examined potential attrition bias since the early school-age follow-ups (Reynolds, 1995; Reynolds, Mehana, & Temple, 1995, 2001), direct adjustments for differential attrition have been modeled in three major studies in adulthood (Reynolds et al., 2011b; Reynolds, Ou, & Temple, 2018; Temple, Reynolds, & Miedel, 2000). Using maximum likelihood (ML) estimation (Greene, 1995) of the joint error term correlation between sample retention and outcome equations, Temple et al. (2000) found that CPC preschool significantly linked to a 6 percentage point lower rate of high school dropout at age 18. This was nearly identical to the 7-point difference in the standard probit model with no attrition adjustment.

Since ML simultaneous equation models rely on the assumption of a valid identifying variable, the CLS began to utilize inverse probability weighting (IPW) techniques starting in 2011. As the most flexible of techniques in which the process of attrition is modeled separately to estimate a predicted probability of sample retention without the assumption of joint multivariate normality across equations (Imbens & Wooldridge, 2009; Kurth et al., 2006), IPW usually includes a large number of predictors of sample recovery. These predictors of sample recovery are used to create weights based on the likelihood that an observation is a member of the recovery sample (not missing outcome data, *R* = 1; if no depressive symptoms data, 0; Seaman & White, 2013). The inverse of the probability of being present in the follow-up sample (having a valid outcome score) is used as the weight variable in a weighted least squares regression or probit regression analysis. IPW methods have been shown to yield the most efficient coefficient estimates (Hirano et al., 2003; Imbens & Wooldridge, 2009) and without strong assumptions about the relation between the determinants of attrition and outcomes (Kurth et al., 2006). On the contrary, regression-based corrections (e.g., as was used in Reynolds & Ou, 2009) assume that the relation between a propensity score and outcome is linear (this is not modeled in weighted regressions with IPW), and there are no specification errors in the model. Multiple imputation of missing outcome data for the attrition sample is also based on the potentially questionable assumption of missing at random—an assumption that is increasingly likely to be violated as the percentage of missing data increases.

The first published CLS study to utilize IPW (Reynolds & Ou, 2011) weighted analyses based on 26 predictors of participant attrition in early adulthood. Results indicated that the effects of CPC preschool on an index of socioeconomic status at age 28 were higher in the IPW attrition model compared to the standard regression model including covariates (a difference of 5.4 points vs. 4.9 points). This pattern was also found for a reduction in felony arrest (4.8 points vs. 4.4 points) as well as other outcomes (e.g., substance abuse). Using the same 26 predictors of sample attrition/recovery and also verifying strong ignorability and common support assumptions, Reynolds et al. (2018) found that the estimated effects of preschool on educational attainment at age 35 were slightly higher for the IPW attrition model compared to the standard regression model. For example, in the IPW model, CPC preschool graduates had a rate of on-time graduation that was 7.1 points higher than the comparison group compared to 6.3 points in the standard model. Similar patterns were also found for several other outcomes. However, to this date, the CLS has not yet applied IPW to analyses of mental health outcomes—an important gap that the present study addresses.

## The Present Study

The present study reanalyzes the original Reynolds and colleagues’ (2007) study for attrition bias in CPC impacts on depressive symptoms using IPW methods. Since prior studies have fully examined potential selection bias into the program (Reynolds, 2008; Reynolds & Ou, 2011; Reynolds & Temple, 1995; Reynolds et al., 2011b), this is not addressed other than as part of robustness testing. We also do not examine attrition bias in the school-age or extended intervention (preschool–third grade). The two major questions are (a) Is the impact of the CPC preschool program on depressive symptoms similar when estimated by IPW methods? and (b) Are IPW estimates of program impact consistent across different model specifications and predictors of attrition?

## Method

### CLS

Data were drawn from the CLS, a prospective investigation that tracks the development of 1,539 individuals who attended early childhood programs in low-income, urban neighborhoods. The original sample was evenly split by sex, 92.9% African American, and 7.1% Hispanic. Intervention group members (*N* = 989) attended the CPC preschool program at age 3 or 4 years old. The program provides comprehensive services to low-income children and families as described in the next section. Comparison group members (*N* = 550) attended the usual kindergarten intervention (full-day programs) in five randomly selected schools or seven CPC schools with 15% attended Head Start preschool. All CLS participants attended kindergarten in 1985–1986. Previous research has confirmed that the two groups are comparable on key individual, family, and school-level characteristics and that the sample is representative of children living in urban poverty (Reynolds & Ou, 2011; Reynolds et al., 2011b). Participant, parent, and teacher surveys as well as school and human service agency records have been collected over the course of the CLS. Table 1 displays the CLS sample characteristics over time.

**Table 1.** Child Characteristics of the Original and Study Samples in the CLS.

Characteristics	Original Sample (*N* = 1,539)	Study Sample (*N* = 1,142)	Attrition Sample (*N* = 397)
Females (%)	50.4	54.3***	39.3
African American (%)	93.0	94.1***	89.7
CPC preschool (%)	64.3	65.7*	60.2
CPC school-age program (%)	55.0	56.4	52.0
Family risk index, ages 0–3 (maximum possible = 8)	4.5	4.5	4.6
Mother under age 18 at participant’s birth (%)	16.2	17.0	13.9
Mother high school dropout (%)	54.3	52.5*	59.2
Mother unemployed or employed part-time (%)	66.3	65.6	68.5
Single-parent household (%)	76.5	75.0*	80.6
Household had four+ children (%)	16.6	17.6	13.9
60% or greater poverty in school attendance area (%)	76.0	76.3	75.1
Family income below 185% of federal poverty line (%)	62.8	61.1*	67.5

*Note*. CLS = Chicago Longitudinal Study; CPC = Child-Parent Centers.

*p < .05. **p < .01. ***p < .001.

### Study Sample

74.2% of the original sample (*N* = 1,142) completed a survey about their life experiences at ages 22–24. This sample is comprised of a slightly higher proportion of males and African Americans than the original CLS sample. Participants who were lost to attrition were more likely to have had mothers who were not high school graduates, to have lived in single-parent households, and to have had family incomes below 185% of the federal poverty line between ages 0 and 3 compared to participants who were not lost to attrition. However, mean risk index scores did not vary significantly by attrition status (Table 1). Notably, low socioeconomic status in childhood has been linked to increased risk of depression in adulthood (e.g., Gilman et al., 2002). Indicative of a simple attrition difference, a greater proportion of the program group was available at follow-up relative to the comparison group (75.8% vs. 71.3%; χ^2^ = 3.8; *p* = .051). The results in Table 1 show that the study sample is not representative of the original sample on salient characteristics, but this does not assess differences in attrition by program and comparison groups.

To assess whether selective attrition is present, a two-way analysis of variance for four baseline attributes was conducted with CPC preschool and sample retention status as main effects (independent variables). A significant program by sample retention interaction would indicate selective attrition. As shown in Table 2, interaction terms for all four baseline characteristics had nonsignificant *F* values. This pattern reveals that the characteristics of participants lost to attrition were similar between groups. In multivariate analysis, however, the accumulation of small differences could be meaningful, as this is not assessed in univariate tests. The difference-in-difference means and effect sizes show that the attrition groups were more disadvantaged on baseline attributes, suggesting a conservative bias if the program has compensatory effects. The main effect results in Table 2 show that both CPC preschool and study participants were more advantaged on several baseline attributes, although for baseline reading achievement, this difference reflects the positive effects of preschool.

**Table 2.** *F* Statistics and Group Differences for Assessing Selective Attrition on Baseline Sample Characteristics.

Model Variables	Family Risk Index	Parent High School Dropout	Female Participant	Baseline Reading Achievement (*K*)
*F* Values				
CPC preschool	0.06	11.1*	0.6	46.4* ^a^
Sample retention status	2.83	5.5*	25.5*	13.0*
CPC × retention status	0.39	0.5	1.4	1.3
Group differences (CPC × retention)				
Difference in difference	0.04	0.043	0.071	1.7
Compar. (lost, retained)	4.7, 4.5	0.67, 0.58	0.39, 0.50	58.7, 60.6
Program (lost, retained)	4.6, 4.5	0.55, 0.50	0.38, 0.56	63.1, 66.8
Effect size of difference (*SD*)	0.02	0.09	0.14	0.13
Difference > for comparison?	Yes	Yes	No	No
High-risk group more affected?	Yes	Yes	Yes	Yes

*Note*. *N* = 1,539; model variables entered simultaneously in a two-way analysis of variance. Test statistic for high school dropout and male is approximated by *F* test. *F* values for early socioemotional adjustment matched those for achievement (15.3, 24.1, and 1.5, respectively). Effect size for difference in difference was .14. CPC = Child-Parent Centers; *SD* = standard deviation.

^a^ As expected, this difference reflects the effect of preschool on end-of-k reading achievement.

*p < .05 and all favor the preschool or sample retention group.

### CPC Program

The CPC program is a preschool to third-grade intervention designed to improve school achievement and performance for children growing up in economically disadvantage neighborhoods or are otherwise at risk of school failure. CLS participants attended the program in 20 schools beginning at age 3 or 4 in 1983–1985 and could continue participating through third grade in 1989. A full description of the program and its history is available (Reynolds, 2000; Reynolds & Mondi, 2016). A matched comparison group of children the same age participated in typically available interventions for children at risk in their areas. Implemented in the Chicago Public School District since 1967 through Title I funding from the Elementary and Secondary Education Act (now Every Student Succeeds Act), CPCs provide comprehensive educational enrichment and family support services beginning at age 3 or 4 in colocated elementary schools.

Core elements reflecting the principles on early enrichment and continuity from preschool to third grade include (a) small classes of no more than 17 preschool children (25 children in K–3, including classroom assistants), (b) Bachelors-level licensed teachers, (c) curriculum focus on language and literacy within a developmental whole-child philosophy, (d) family support services through workshops, education, and training events in the parent resource room, and (e) coordination of instruction across grades by a leadership team in each site (head teacher, parent resource teacher, and school-community representative). The latter staff member conducts home visits and mobilizes resources in the school community for parents (economic or housing assistance, health and mental health services, and employment opportunities). Health, nutrition, speech therapy, and auxiliary services are also provided (Reynolds, 1994; Reynolds et al., 2003).

### Outcome Measure

#### Depressive symptoms

As part of a survey on education and well-being, participants completed a five-item modified version of the depressive subscale of the Brief Symptom Inventory (BSI; Derogatis, 1975) at ages 22–24. Participants were asked “During the last month, have you felt” the following, and “If Yes”…how often have you felt this way?”:

(a) depressed
(b) hopeless
(c) lonely
(d) life isn’t worth living
(e) very sad.

Responses ranged from 0 (*not at all*) to 5 (*almost every day*) with the middle category of 3 (*about once per week*). The sixth item “Have you felt anxious?” was not sufficiently correlated with the other items. The reliability of the scale was .84. Based on these items, an overall dichotomous variable was created, reflecting the frequency and severity of participants’ symptoms. They were coded as 1 if they felt depressed, lonely, or sad “almost every day,” hopeless at least “a few times per week,” or life is not worth living “at least two or three times a month.” All others were coded 0. The BSI does not measure clinical depression; however, individuals who report significant symptoms on the BSI are likely at increased risk for major depressive disorder. The use of a dichotomous variable is consistent with previous BSI research and is intended to prevent case overidentification (Derogatis & Lynn, 2000; Shrout & Yager, 1989).

Eight participants completed the survey but did not fill out the depression index. Based on their responses to depression items in high school and responses to questions about life satisfaction, optimism, and mental health treatment history on the adult survey, we gave each participant a code of “0” for adult depressive symptoms, resulting in a study sample size of 1,142.

### Predictors of Attrition/Sample Retention for IPW Propensity

Thirty-two variables were included in the model predicting participants’ probability of being in the adult follow-up sample: (1) CPC preschool participation; (2) school-age CPC participation; (3) sex; (d) African American; (4) low birth weight; (5) word analysis skills at the end of kindergarten; (6) composite school readiness score; (7) substantiated maltreatment between ages 0 and 3; (8) participant’s mother was not a high school graduate by participant age 3; (9) participant was eligible for free lunch between ages 0 and 3; (10) participant’s mother was under age 18 at the participant’s birth; (11) participant lived in a household of four or more children between ages 0 and 3; (12) participant’s family income was below 185% of the federal poverty level between ages 0 and 3; (13) participant’s mother was unemployed or employed part-time when participant was between ages 0 and 3; (14) participant lived in a single-parent household between ages 0 and 3; (15) a dichotomous variable indicating that information was not available about ages 0–3 risk indicators (Items 8–14 above); (16) participant lived in a school attendance area where at least 60% of households were impoverished; (17) frequent family conflict between ages 0 and 5; (18) family financial problems between ages 0 and 5; (19) parental substance abuse problems between ages 0 and 5; (20) participant was active in Chicago Public Schools for at least 6 years between kindergarten and eighth grade; (21) participant’s mother participated in at least 2 years of postsecondary education by participant age 17; (22) number of school moves between kindergarten and Grade 4; (23–26) variables indicating the percentage of individuals living 1 year, between 1 and 5 years, 5–10 years, or 10–20 years, respectively, within the participants’ housing unit by age 4; (27) percentage of self-employed individuals aged 16 and older within the participant’s census tract by age 4; (28) percentage of female-headed black households within the participant’s census tract by age 4; (29) magnet school attendance between fourth and eighth grade; (30) eighth-grade reading score; (31) participant was arrested as a juvenile; (32) the CLS located a Social Security number for the participant by 2007.

### Covariates in the Outcome Model

Covariates included sex, race/ethnicity, low birth weight, CPC preschool participation, CPC school-age participation, family risk index (ages 0–3); family conflict, substance abuse of a parent, and family financial problems (ages 0–5), survey completion date, and survey mode (in person or by mail). See Online Appendix for full variable descriptions.

### Statistical Analyses

IPW is estimated independently of the outcome specification model and utilizes all available data to estimate complex adjustments. Previous research has demonstrated that the IPW approach yields lower variances and standard errors in large study samples than other propensity methods (Imbens & Wooldridge, 2009). Propensity score approaches can be used to help limit bias that arises from differences in observable characteristics (Rosenbaum & Rubin, 1983 **)** in quasi-experimental approaches. Given the minor differences in the treatment and control group in the CLS sample and that results do not fundamentally change when weighting by program selection and attrition probabilities, this analysis focuses on minimizing differences that arise from differences in attrition weights.

In the IPW approach, logit regression is conditioned on a set of predictors (*X*) that are hypothesized to influence participants’ probability of being in the recovery sample, yielding a predicted probability of being in the recovery sample (*R* = 1), where

1 $$\text{Pr}\left(R_{i}=1\text{|}X,Z\right)={\text{ β}}_{1}X_{i}+{\text{ β}}_{2}Z_{i}+e_{i},$$

and the weight is calculated as

wi= 1Pr(Ri=1|X,Z) for Ri=1 

and

wi= 1(1−Pr(Ri=1|X,Z)) for Ri=0.

The predicted probabilities of sample retention (no attrition) were derived from logit regression with the 32 input variables described earlier. The difference in predicted probabilities between groups weighted for these sample retention propensities is the estimated program effect. The fit of the propensity model was determined by whether the program and comparison groups were balanced on the covariates after weighting. Coefficients were transformed to marginal effects in percentage points. Robust standard errors for school clustering were based on the Huber-White method; 95% confidence intervals are reported along with *p* values of .05 (two-tailed tests) to denote statistical significance.

The IPW regression analysis was conducted in STATA (Version 15). We estimated the effects of CPC preschool on the prevalence of depressive symptoms at ages 22–24 with probit regression. Given the reanalysis focus, we did not investigate other measures of depression. Missing data on the predictors and covariates, which ranged from 5% to 20%, were imputed using the expectation–maximization algorithm. Effect sizes (Cohen’s *d*) were computed from the marginal effects via the probit transformation of proportions. Values of .20 standard deviations (*SDs*) or higher in absolute value were considered practically significant.

## Results

Findings are organized into three sections. We first assess the quality and fit of the propensity score model in adjusting for potential attrition bias. Main impact findings are then presented for the estimated model in Equation 1 and several alternatives. Robustness testing for consistency of impact estimates is also reported. The Online Appendix describes these and additional findings.

### Predictors of Sample Retention for the IPW Model

The primary logistic regression model was drawn from previous CLS studies predicting attrition (Arteaga et al., 2014; Reynolds et al., 2011b) and depressive symptoms (Mondi et al., 2017). The results of this model are displayed in Table 3 with marginal effects in percentage points. Of the 32 predictors, 14 were significant at the .05 level. Among the demographic variables, female participants were more likely to be the retention sample as were those in higher income families and from married households. The sample retention rate for females, for example, was 14.1 percentage points higher than males above and beyond other predictors. CPC participation was not associated with sample retention. Significant behavioral predictors, most of which favored more advantaged participants in the retention sample, included word analysis skills in kindergarten, school moves between kindergarten and fourth grade, and juvenile arrest. For the latter, youth who were arrested were more likely to be in the retention sample. This is partly due to a focus in the adult survey on locating and interviewing those in the justice system.

**Table 3.** Predictors of CLS Sample Retention.

Variable	Marginal Effect (*dy*/*dx*)	Standard Error
CPC preschool	0.0145	.0262
CPC school-age participation	−0.0232	.0256
Kindergarten word-identification score	0.0017*	.00102
Composite kindergarten school readiness score	0.00289*	.00167
Substantiated maltreatment (ages 0–3)	−0.0439	.0626
Mother was not a high school graduate (by participant aged 3)	−0.0276	.0263
Eligible for free lunch (ages 0–3)	−0.00194	.0343
Mother was under 18 at participant’s birth	0.0846***	.0272
Lived in a household of four or more children (ages 0–3)	0.0321	.0301
Family income below 185% of the federal poverty level (ages 0–3)	−0.0755**	.0332
Mother was unemployed or employed part-time (ages 0–3)	0.0528	.0362
Lived in a single-parent household (between ages 0 and 3)	−0.0690***	.0264
Lived in a school attendance area where at least 60% of households were impoverished (ages 0–3)	0.0730**	.0369
Mother participated in at least two years of postsecondary education (by participant aged 17)	0.0407	.0251
Information is missing for at least one age 0–3 risk indicator	−0.0548	.0381
Lived in a school attendance area where at least 60% of households were impoverished (ages 0–3)	−0.0245	.0264
Low birth weight	0.0313	.0324
Frequent family conflict (ages 0–5)	−0.00246	.0573
Family financial problems (ages 0–5)	0.0470	.0441
Parental substance abuse (ages 0–5)	−0.00828	.0638
Female	0.141***	.0239
African American	0.0949*	.0532
Number of school moves between Kindergarten and Grade 4	−0.0226*	.0137
Social Security number identified by 2007	0.248***	.0670
% of individuals living 1 year within the participant’s housing unit (by participant aged 4)	−1.082***	.370
% of individuals living 1–5 years within the participant’s housing unit (by participant aged 4)	−0.573	.365
% of individuals living 5–10 years within the participant’s housing unit (by participant aged 4)	−0.508	.345
% of individuals living 10–20 years within the participant’s housing unit (by participant aged 4)	−0.711**	.345
% of self-employed individuals ages 16+ within the participant’s census tract (by participant aged 4)	0.833	.785
% of female-headed black households within the participant’s census tract (by participant aged 4)	−0.142*	.0841
Eighth-grade reading score	0.000575	.000647
Juvenile arrest	0.0866***	.0234
Magnet school attendance (Grades 4–8)	0.00518	.0105
Observations	1,516	

*Note*. CLS = Chicago Longitudinal Study; CPC = Child-Parent Centers.

*p < .1. **p < .05. ***p < .01.

Given these results, Table 4 shows the standardized mean differences between CPC preschool and comparison groups in the rate of sample retention before and after weighting by the IPW attrition estimate from the logit model. For the sample retention prediction model to be effective, standardized mean differences (effect sizes) should be less than .25 *SD* across the included covariates. If the distribution (balance) of each of the covariates between groups was identical—completely overlapping—effect sizes after weighting would be zero. Table 4 shows that for nearly all covariates, effect sizes were between 0 and .05 in absolute value. This indicated that after weighting on the IPW attrition propensity, preexisting group differences in attrition attributes were eliminated. This pattern supports the strength of the propensity model. The largest difference was for teenage parenthood, as a greater proportion of retained participants in CPC were less likely to parents under age 18. This difference, however, was not significant.

**Table 4.** Standardized Mean Differences Pre- and Postweighting on the Attrition Propensity Score for Depressive Symptoms, With Age 0–5 Composite Risk Index, Year, and Mode of Survey Completion.

Predictor of Propensity Score	Preweight Standard Difference	Postweight Standard Difference
CPC preschool program	.089	.029
CPC school-age program	.071	.032
Kindergarten word-identification score	.244	−.002
Composite school readiness score	.289	−.037
Any child welfare cases	−.05	−.001
Mother did not complete high school	−.118	−.004
Eligible for free lunch	−.055	.035
Mother less than 18	.076	−.069
Four or more children	.066	.043
TF participation	−.122	.024
Mother unemployed	−.05	.036
Single parent	−.116	.024
Active 6 years in CPS	.31	.021
Mother reports postsecondary education	.241	−.046
Missing risk indicator data	−.286	.028
60% or greater poverty in school area	.007	−.002
Low birth weight	.021	−.02
Frequent family conflict (ages 0–5)	.016	.023
Family financial problems (ages 0–5)	.013	−.023
Parental substance abuse (ages 0–5)	0	.001
Female	.347	−.025
African American	.164	−.022
Number of school moves between kindergarten and Grade 4	−.184	.02
Social Security number identified by 2007	.357	−.012
% individuals living 1 year within the participant’s housing unit (by participant aged 4)	−.154	.064
% individuals living 1–5 years within the participant’s housing unit (by participant aged 4)	−.048	−.009
% individuals living 5–10 years within the participant’s housing unit (by participant aged 4)	.135	−.035
% individuals living 10–20 years within the participant’s housing unit (by participant aged 4)	.013	−.028
% self-employed individuals ages 16+ within census tract (by participant aged 4)	.06	.005
% female-headed Black households within participant’s census tract (by participant aged 4)	−.105	.005
Eighth-grade reading score	.218	.017
Any juvenile arrest	.077	−.009
Magnet school attendance (Grades 4–8)	.156	−.016

*Note.* CPC = Child-Parent Centers; CPS = Chicago Public Schools; TF = Temporary Assistance for Needy Families.

### Impact Estimates of CPC and Depressive Symptoms

From the model above, the weighted impact estimates from probit regression are summarized in Table 5. Prior to IPW attrition adjustment, the unadjusted marginal effect of CPC preschool was −4.8 percentage points (*p* = .036; Model 1) with those from prior studies showing impacts in range of −4.6 to – 4.9 (Models 2 and 3), including that from the 2007 study. The covariates-only model in the reanalysis resulted in a difference of −5.2 percentage points (Model 4; *p* = .036), a 28% reduction in symptoms over the comparison group.

**Table 5.** Estimated Effects of CPC Preschool Participation for Depressive Symptoms (1+) by Model Specification and Across Studies.

Model	Analytic Method and Study	Program Comparison in Percentage Points [95% CI] (*p* Value)	Reduction Over Observed Comparison Group Rate of 18.4%	Effect Size (Cohen’s *d*)
1	Unadjusted model	−4.8[−9.2, −0.3] (.037)	26%	−.21
2	Reynolds et al. (2007) model with demographic^a^ and age 0–3 risk indicator^b^ covariates	−4.6[−9.5, 0.3] (.057)	25%	−.20
3	Reynolds et al. (2007) model with demographic^a^ and age 0–3 risk indicator^c^ covariates plus propensity score for attrition	−4.9[−5.4, −4.3] (.040)	27%	−.22
4	Present model with demographic,^a^ low birth weight, age 0–3 risk index,^b^ and age 0–3 family risk indicator^c^ covariates	−5.2[−10.1, −0.4]	28%	−.25
5	Model 4 with IPW attrition adjustment	−7.1[−7.8, −6.5]	39%	−.32
6	Model 4 with IPW program selection adjustment	−7.3[−7.9, −6.6]	40%	−.32
7	Model 4 with IPW attrition and program selection adjustments	−7.9 [−8.6, −7.2]	43%	−.34

*Note*. CPC = Child-Parent Centers; IPW = inverse probability weighting.

^a^ Female, African American.

^b^ Composite of eight age 0–3 risk indicators.

^c^ Mother was under age 18 at the participant’s birth, mother was not a high school graduate, mother was unemployed or employed part-time, participant lived in a single-parent household, participant lived in a household of four or more children, participant lived in a school attendance area where at least 60% of households were impoverished, participant’s family income was below 185% of the federal poverty level, participant was eligible for free lunch. Not shown is the Reynolds et al. (2007) estimates using the family risk index instead of the individual risk indicators: −6.5 points (95% CI = [−5.9, −7.1]).

Model 5 is the IPW attrition-adjusted model and shows a marginal effect of −7.1 percentage points (95% CI = [−9.7, −5.4]; *p* < .001) in favor of the CPC group. This impact, while within the confidence interval of the original study (95% CI = [−9.5, 0.3]), is 54% higher than the −4.6 percentage point value for the original study. The effect size increased from −0.20 to −0.32. The reanalyzed estimate is interpreted as the impact of the CPC preschool program after adjusted for the influence of baseline covariates and each sample member’s propensity to remain in the adult follow-up sample. The relatively narrow confidence interval indicates increased precision in the estimated effect since the standard error of the estimate is smaller than other estimates. Notably, however, the validity of the estimate is based on the adequacy of the propensity model in predicting attrition and the assumption of no unmeasured confounders that if included in the model would uniquely predict depressive symptoms or program-related attrition. For further comparison, Models 6 and 7 include adjustments for program selection. Model 7, which includes the double adjustment of attrition and program selection, has a larger marginal effect (−7.9 percentage points; *p* < .001). This indicates a synergy of sample selection influences that strengthen program effects.

Table 6 shows the other predictors of depressive symptoms in the impact model. Among the CPC group, Black participants, participants affected by high levels of family risk between ages 0 and 3, and participants who experienced family financial problems before age 5 were more likely to endorse significant depressive symptoms by age 24. Among the comparison group, Black participants and participants who participated in the school-age CPC program were more likely to endorse significant depressive symptoms, and females were less likely to endorse symptoms. Participants affected by parental substance abuse before age 5 were less likely to endorse symptoms. Among the comparison group, Black participants and participants who participated in the school-age CPC program were more likely to endorse depressive symptoms at ages 22–24. Year and mode of survey completion (in person or via mail) were not significantly associated with self-reported symptoms (see Online Appendix for additional results). The mean IPW attrition-adjusted marginal effect is shown at the bottom of Table 6.

**Table 6.** Regression Model Predicting Depression Symptoms at Ages 22–24, With Ages 0–5 Composite Risk Index, Including Year and Mode of Survey Completion, With IPW for Ages 22–24 Attrition.

Predictor	CPC Preschool Group		Comparison Group	
	*dy*/*dx*	95% CI	*dy*/*dx*	95% CI
CPC school-age participation	−.03	[−0.08, 0.03]	.11**	[0.02, 0.21]
Black	.10***	[0.04, 0.15]	.12**	[0.03, 0.21]
Female	.03	[−0.02, 0.07]	−.06	[−0.14, 0.13]
Low birth weight	−.04	[−0.10, 0.02]	.02	[−0.10, 0.13]
Family risk (ages 0–3)	.02**	[0.01, 0.04]	.01	[−0.01, 0.03]
Family conflict (ages 0–5)	.10	[−0.02, 0.23]	.12	[−0.10, 0.35]
Parental substance abuse (ages 0–5)	−.07*	[−0.13, −0.01]	.08	[−0.24, 0.41]
Family financial problems (ages 0–5)	.20**	[.07, 0.32]	−.01	[−0.18, 0.15]
Survey completed in 2002 (ages 22–24)	−.01	[−0.07, 0.04]	−.06	[−0.15, 0.03]
Survey returned via mail (ages 22–24)	.10	[0.00, 0.21]	.13	[−0.03, 0.29]
Survey completed in-person (ages 22–24)	−.01	[−0.09, 0.06]	.01	[−0.11, 0.13]
Mean point difference in % of 1+ depression symptoms (ages 22–24)	7.1 points (CI [6.5, 7.8] points)			

*Note*. CPC = Child-Parent Centers; IPW = inverse probability weighting.

**Figure 1.**
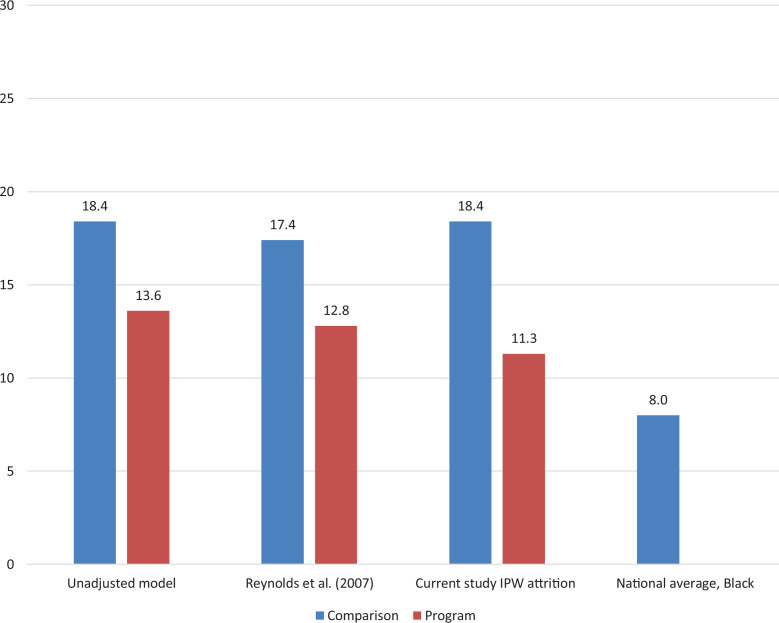
Adjusted rates of depressive symptoms in adulthood for three Child-Parent Centers preschool and *comparison group contrasts*. *Note*. Percentages for the current study are based on the models in Tables 4 and 5. *p* Values for the group differences are .038, .057, and .011, respectively. The unadjusted model includes no covariates or adjustments. National average is for U.S. Black adults from the National Health and Nutrition Examination Survey (NHANES) 2005–2006, using a Patient Health Questionnaire (PHQ) score of 10 or higher. The incidence rates (over the past 2 weeks) for all adults and those below the federal poverty line were 4.7% and 11%, respectively.

Overall, these models’ prediction patterns for depressive symptoms are consistent with previous analyses (Mondi et al., 2017) despite the larger differences in CPC impacts. As summarized in Figure 1, the marginal effect of CPC preschool in the current study is substantially larger than in the 2007 study. Also shown is that the proportion of program and comparison group participants with depressive symptoms is greater than the national average for Black adults, based on data from the National Health and Nutrition Examination Survey (Pratt & Brody, 2008). Given that the study sample grew up on high-poverty neighborhoods, this difference would be expected. It is possible that this national estimate of 8% is conservative given the 2-week reporting interval compared to our study’s 1-month time frame. Nevertheless, the CPC program appeared to substantially reduce the incidence of depressive symptoms in adulthood.

### Robustness

To determine whether the marginal effect using the IPW attrition adjustment (Model 5 in Table 5) was stable to different assumptions, we examined a wide range of alternative models (see the Online Appendix). This included (a) predictor specifications of sample retention propensities, (b) covariate specifications for the outcome model, and (c) IPW adjustment models. Results indicated a high degree of consistency in impact estimates. Marginal effects were unaffected in alternative logit regression models predicting sample retention or covariate specifications in the outcome model. Following previous studies, the double-IPW adjustment in which the propensity weights for attrition is multiplied by the propensity weights for program selection also yielded a similar estimate. If the propensity scores are valid and no unmeasured causes are plausible, this would be expected to be most representative of the true effect of the program.

We further tested through Monte Carlo simulation the statistical differences between the present results and the original 2007 study. The results are reported in Online Appendix Table B7. Based on 10,000 iterations, the mean group difference in percentage points was −7.87 (95% CI = [−7.84, −7.90]; *SD* = 1.48). In only 15.2% of the tests did the estimated main effect fall outside the confidence interval of the 2007 study. This indicates that, at least statistically, the main effect between the two studies is equivalent. However, in terms of practical significance and effect size, the reanalysis yields a substantially larger impact (from 4.6 to 7.9 percentage points). This is most likely due to attrition from the sample.

## Discussion

There is a critical need for large-scale interventions that will reduce the social and economic burden of depression. ECE programs have shown promise as an effective prevention strategy (McLaughlin et al., 2007; Palfrey et al., 2005; Reynolds & Ou, 2011), but scaling to populations has been limited. A previous study by Reynolds et al. (2007) of the CPC preschool program found a 4.6 percentage point reduction in depressive symptoms in early adulthood relative to a matched comparison group (12.8% vs. 17.4%; a 26% reduction over the comparison group). However, the study did not directly address potential attrition biases and achieved only borderline levels of statistical significance.

The present study reanalyzed the impact of CPC preschool for the same measure of depressive symptoms utilizing IPW methods to more robustly address potential attrition bias. Using a propensity score for sample retention estimated with 32 predictors in a logistic model, we found that CPC preschool was linked to a 7.1 percentage point reduction in depressive symptoms after weighting by the propensity score (Figure 1). This is an increase of 54% in estimated impact over the original study and a 60% increase in effect size as calculated by Cohen’s *d* (−0.20 to −0.32). Year and mode of survey completion did not significantly predict symptomology. Although this IPW-adjusted impact is within the confidence interval of the previous study (95% CI = [−9.5 to 0.3]), and for most iterations in the Monte Carlo simulations, the marginal effect may have been substantially underestimated. Indeed, our original estimate from the 2007 study is outside the confidence interval of the IPW-adjusted effect. This suggests imprecision in our estimated model.

One practical consequence of the larger effect on depression in the reanalysis is that it increases the cost savings associated with treatment and lost productivity in the CPC cost-benefit analysis (Reynolds et al., 2011a). The increased savings per participant is USD$332 in net present value (2020 dollars), which are conservative since suicide-related risks and duration of treatment and lost productivity were limited to 2 years. The increased savings would also be expected to increase the benefits of the total program from preschool to third grade, as this also was linked to lower depression. The costs of treatment and lost productivity per person with depression was estimated at USD$13,330 (converted to 2020 dollars; Reynolds et al., 2011a). Using the effect estimated our reanalysis, the increased cost savings for depression represents about 3% of the program cost per participant and substantially higher than this after accounting for broader social benefits and program experiences.

Given the remaining imbalance of some model variables in Table 4, it is possible that we overestimated the marginal effect of CPC participation. However, the magnitude of the difference between the present study’s estimates and those of previous studies and the relative robustness of findings across multiple model iterations support the interpretation that Reynolds et al.’s (2007) study did not fully account for the effects of attrition on depressive symptoms. We also note that both the present study’s outcome covariates and predictors of sample retention were more comprehensive than previous CLS studies, which may have contributed to the increased effect. CPC participants grew up in the highest poverty neighborhoods, and larger effects would be expected by further accounting for early adversities in home and school environments (e.g., family conflict, school quality), especially given the evidence that CPC impacts are compensatory (Reynolds et al., 2001, 2011a,b). Depression and depressive symptoms reflect functional impairments that affect day-to-day living rather than status measures such as education or income. Consequently, adjustment for attrition would be expected to be greater.

Our finding of a larger effect size after IPW attrition correction is further supported in studies of other measures of well-being. Compared to no correction, Reynolds et al. (2018) found that the IPW attrition model increased the estimated effect of CPC preschool on high school graduation by 19% (5.2–6.2 percentage points). For earned associates’ degree or higher by age 35 years, the increase was 13% (4.6–5.2 percentage points). Precision and statistical significance also increased. Using a similar modeling approach, Reynolds et al. (2011) also found in the CLS that estimated preschool effects for substance abuse, a moderate SES level, and health insurance coverage increased by 12%, 8%, and 21%, respectively, under IPW attrition adjustment compared to the standard covariate model. A similar pattern was also found for the impacts of Tulsa universal pre-K on seventh-grade school performance (Gormley et al., 2018). Compared to the main model without IPW adjustment, estimates of program impact with IPW attrition adjustment increased by 8%, 19%, and 29%, respectively, for math achievement, reading achievement, and reductions in grade repetition. Precision and statistical significance also increase after adjustment.

One interpretation is that the attrition weighting is accounting for, at least partly, missing comparison group participants who are not to be fully represented in estimates of social programs. Because they tend to the highest rates of attrition and the worst outcomes on average, adjustments would be expected to be sizable. Certainly, the degree of change in estimates will depend on the amount of missing data, the strength of the propensity score model, and the characteristics of those who attrit. The outcome itself and the source of measurement also may matter, as self-reports of functional competencies, such as in our study, may be prone to larger attrition biases.

Nevertheless, there is currently no consensus about the best approach to addressing attrition and sample selection bias. For example, in the Fast Track prevention program, a socioemotional learning and parent training program beginning in first grade, Jones et al. (2010) found that the intervention reduced the need for outpatient mental health services at the end of high school, 12 years after the intervention began. These estimates were based on multiple imputation of missing outcome data, which at 20%–30% was similar to the CLS study. Also similar to Reynolds et al.’s (2007) previous study, the age 21 follow-up of the Seattle Social Development Program (Hawkins et al., 2005) found a reduced number of depressive symptoms among full-intervention participants which was marginally significant, with missing data addressed in preliminary analysis showing similarity among intervention and comparison groups on baseline attributes. As in the earlier CLS analysis, none of the studies directly assessed alternative approaches.

As noted by Seaman and White (2013), there are advantages to both the IPW approach (as presented in the present article) and the multiple imputation approach to addressing missing data. IPW methodology requires estimating the probability that an observation in the data set has complete data. In this case, it requires a model to predict the probability of having data on adult depressive symptoms. Meanwhile, MI requires a model for the distribution of missing values. Seaman and White (2013) explain that because MI fills all missing data, it can be more a more efficient approach to missing data. IPW approaches can yield larger standard errors if the probability weights generated have a wide distribution. In our case, the weights have a relatively narrow distribution (1.02–33.6). This strengthened confidence in our model estimates.

Using the IPW approach does not require making assumptions of the missing data drawn from complete data that the MI approach requires. Another key advantage of the IPW approach is that it allows us to examine the quality of the missingness model. Finally, Seaman and White (2013) note that the best results may combine MI and IPW methodologies, which can draw on the advantages of both. In the present article, we filled missing demographic control variables using MI, where the missing and nonmissing groups may be more similar, given the nature of the CLS sample. However, we addressed missing data on the depressive symptoms outcome data with the IPW approach, avoiding making assumptions of those missing that data from those observations with complete outcome data.

Although statistical techniques such as IPW and multiple imputation are powerful tools for addressing sampling and attrition issues in longitudinal studies, they are not adequate substitutes for robust study design and participant retention strategies. Participant retention strategies may be particularly important in studies of participants affected by high levels of sociodemographic risk, who may be more difficult to maintain contact with over time. The present study, which reinterviewed young adult participants who were enrolled in the study as kindergarteners, utilized a variety of methods to maintain contact with participants over the years (e.g., mailings, contacting family members and friends, online database searches, coordinating with schools), resulting in an unusually high sample retention rate of 74% in adulthood.

Since attrition continues to be a major data collection and analytic challenge in longitudinal evaluation studies, the need to directly incorporate correction strategies in impact assessment is high. This is especially true when there is larger sample loss and evidence that attrition is selective. Large-scale studies, even if they have substantial resources, face many hurdles to successful follow-up. Response rates in 2-year follow-ups are often as low as 50% and are typically lower over a decade or more. Historically, adjustments for potential attrition bias have not been part of impact analysis. Rather, attrition comparisons of those missing and not missing have been compared to determine whether representativeness is maintained and selective attrition is avoided. Early childhood cohort studies tracking samples in excess of 200 have observed generally lower rates of sample recovery than the CLS. Leading examples include the Infant Health and Development Program (65% up to age 18; McCormick et al., 2006), Houston Parent-Child Development Center (63% up to age 18; Johnson & Blumenthal, 2004), National Early Head Start Impact Study (55% up to age 5; Chazan-Cohen et al., 2007), and the Consortium for Longitudinal Study (1983; 55% up to age 22). The IPW strategy applied in the present study, multiple imputation, and a combination of the two would be informative in assessing the consistency of findings across a range of studies and intervention approaches. Of course, strategies to improve response rates in contemporary studies and in the future are needed.

## Conclusion

Depression exerts significant burdens on individuals and society and disproportionately affects low-income individuals. CPC and similar programs lay important foundations for lifelong well-being, including mental health. Program scale-up is dependent on accurate estimation of program effects. Utilizing contemporary methods of attrition adjustment via propensity score weighting, the present investigation found a stronger effect of CPC preschool on adult depressive symptoms than in the original study. The magnitude of increase was large and of practical significance, though within the confidence interval of the original study. These results strengthen the evidence that large-scale, high-quality ECE programs can promote long-term mental health. Continued investigation of alternative strategies of accounting for sample selection and attrition biases is needed as is process of change by which prevention programs lead to long-term benefits in mental health and well-being.

## Supplemental Material

Supplemental Material, sj-docx-1-erx-10.1177_0193841X20976527 - Early Childhood Education and Adult Depression: An Attrition Reanalysis With Inverse Propensity Score Weighting Click here for additional data file. Supplemental Material, sj-docx-1-erx-10.1177_0193841X20976527 for Early Childhood Education and Adult Depression: An Attrition Reanalysis With Inverse Propensity Score Weighting by Christina F. Mondi, Arthur J. Reynolds and Brandt A. Richardson in Evaluation Review

## References

[bibr1-0193841X20976527] Arteaga I. Humpage S. Reynolds A. J. Temple J. A. (2014). One year of preschool or two—Is it important for adult outcomes? Results from the Chicago Longitudinal Study of the Child-Parent Centers. Economics of Education Review, 40, 221–237. 26823640 10.1016/j.econedurev.2013.07.009 PMC4727175

[bibr50-0193841X20976527] Barry A. E. (2005). How attrition impacts the internal and external validity of longitudinal research. The Journal of School Health, 75(7), 267–270. 10.1111/j.1746-1561.2005.00035.x 16102089

[bibr51-0193841X20976527] Carkin D. M. Tracy P. E. (2015). Adjusting for unit non-response in surveys through weighting. Crime & Delinquency, 61(1), 143–158. 10.1177/0011128714556739

[bibr3-0193841X20976527] Chazan-Cohen R. Ayoub C. Pan B. A. Roggman L. Raikes H. Mckelvey L. Whiteside-Mansell L. Hart A. (2007). It takes time: Impacts of Early Head Start that lead to reductions in maternal depression two years later. Infant Mental Health Journal, 28, 151–170. 28640556 10.1002/imhj.20127

[bibr5-0193841X20976527] Derogatis L. R. (1975). The brief symptom inventory. Clinical Psychometric Research.

[bibr6-0193841X20976527] Derogatis L. R. Lynn L. L. (2000). Screening and monitoring psychiatric disorder in primary care populations. In Maruish M. E. (Ed.), Handbook of psychological assessment in primary care settings (pp. 115–152). Routledge.

[bibr7-0193841X20976527] Fitzgerald J. Gottschalk P. Moffitt R. (1998). An analysis of sample attrition in panel data: The Michigan Panel study of Income Dynamics. The Journal of Human Resources, 33(2). 10.2307/1446433

[bibr8-0193841X20976527] Gilman S. E. Kawachi I. Fitzmaurice G. M. Buka S. L. (2002). Socioeconomic status in childhood and the lifetime risk of major depression. International Journal of Epidemiology, 31(2), 359–367. 10.1093/ije/31.2.359 11980797

[bibr9-0193841X20976527] Gormley W. T. Phillips D. Anderson S. (2018). The effects of Tulsa’s pre-K program on middle school student performance. Journal of Policy Analysis and Management, 37(1), 63–87.

[bibr10-0193841X20976527] Greenberg P. E. Fournier A. A. Sisitsky T. Pike C. T. Kessler R. C. (2014). The economic burden of adults with major depressive disorder in the United States (2005 and 2010). Journal of Clinical Psychiatry, 76(2), 155–162. 10.4088/JCP.14m09298 25742202

[bibr52-0193841X20976527] Greene W. H. (1995). LIMDEP (Version 7) [Computer software]. Econometric Software, Inc.

[bibr11-0193841X20976527] Härkänen T. Kaikkonen R. Virtala E. Koskinen S. (2014). Inverse probability weighting and doubly robust methods in correcting the effects of non-response in the reimbursed medication and self-reported turnout estimates in the ATH survey. BMC Public Health, 6(14), 1150. 10.1186/1471-2458-14-1150 PMC4246429 25373328

[bibr12-0193841X20976527] Hawkins J. D. Kosterman R. Catalano R. F. Hill K. G. Abbott R. D. (2005). Promoting positive adult functioning through social development intervention in childhood: Long-term effects from the Seattle Social Development Program. Archives of Pediatrics and Adolescent Medicine, 159, 25–31. 15630054 10.1001/archpedi.159.1.25

[bibr13-0193841X20976527] Heckman J. J. (2011). The economics of inequality: The value of early childhood education. American Educator, 35(1), 31–47.

[bibr14-0193841X20976527] Hirano K. Imbens G. W. Ridder G. (2003). Efficient estimation of average treatment effects using the estimated propensity score. Econometrica, 71, 1161–1189. 10.1111/1468-0262.00442

[bibr15-0193841X20976527] Imbens G. W. Wooldridge J. M. (2009). Recent developments in the econometrics of program evaluation. Journal of Economic Literature, 47, 5–86. 10.1257/jel.47.1.5

[bibr16-0193841X20976527] Johnson D. L. Blumenthal J. (2004). The parent child development centers and school achievement: A follow-up. The Journal of Primary Prevention, 25, 195.

[bibr17-0193841X20976527] Jones D. Godwin J. Dodge K. A. Bierman K. L. Coie J. D. Greenberg M. T. Lochman J. E. McMahon R. J. Pinderhughes E. E. (2010). Impact of the fast track prevention program on health services use by conduct-problem youth. Pediatrics, 125(1), e130–e136. 20008428 10.1542/peds.2009-0322 PMC3534731

[bibr18-0193841X20976527] Kessler R. C. Berglund P. Demler O. Jin R. Merikangas K. R. Walters E. E. (2005). Lifetime prevalence and age-of-onset distributions of DSM-IV disorders in the National Comorbidity Survey Replication. Archives of General Psychiatry, 62(6), 593–602. 10.1001/archpsyc.62.6.593 15939837

[bibr19-0193841X20976527] Kurth T. Walker A. M. Glynn R. J. Chan K. A. Gaziano J. M. Berger K. Robins J. M. (2006). Results of multivariable logistic regression, propensity matching, propensity adjustment, and propensity-based weighting under conditions of nonuniform effect. American Journal of Epidemiology, 163, 262–270. 10.1093/aje/kwj047 16371515

[bibr20-0193841X20976527] McCormick M. C. Brooks-Gunn J. Buka S. L. Goldman J. Yu J. Salganik M. Scott D. T. Bennett F. C. Kay L. L. Bernbaum J. C. Bauer C. R. Martin C. Woods E. R. Martin A. Casey P. H. (2006). Early intervention in low birth weight premature infants: Results at 18 years of age for the infant health and development program. Pediatrics, 117, 771–780. 16510657 10.1542/peds.2005-1316

[bibr21-0193841X20976527] McLaughlin A. E. Campbell F. C. Pungello E. P. Skinner M. (2007). Depressive symptoms in young adults: The influences of the early home environment and early educational childcare. Child Development, 78(3), 746–756. 10.1111/j.1467-8624.2007.01030.x 17517002

[bibr22-0193841X20976527] Mondi C. F. Reynolds A. J. Ou S. (2017). Predictors of depressive symptoms in emerging adulthood in a low-income urban cohort. Journal of Applied Developmental Psychology, 50, 45–59. 10.1016/j.appdev.2017.03.009 28936020 PMC5602590

[bibr23-0193841X20976527] Ou S. R Reynolds A. J. (2006). Early childhood intervention and educational attainment: Age 22 findings from the Chicago Longitudinal Study. Journal of Education for Students Placed at Risk, 11(2), 175–198.

[bibr24-0193841X20976527] Palfrey J. S. Hauser-Cram P. Bronson M. B. Warfield M. E. Sirin S. Chan E. (2005). The Brookline Early Education Project: A 25-year follow-up study of a family-centered early health and development intervention. Pediatrics, 116(1), 144–152. 10.1542/peds.2004-2515 15995045

[bibr25-0193841X20976527] Pratt L. A. Brody D. J. (2008). Depression in the United States household population, 2005-2006 (NCHS data brief No. 7). National Center for Health Statistics, U. S. Department of Health and Human Services.

[bibr26-0193841X20976527] Reynolds A. J. (1994). Effects of a preschool plus follow-on intervention for children at risk. Developmental Psychology, 30(6), 787–804. 10.1037/0012-1649.30.6.787

[bibr54-0193841X20976527] Reynolds A. J. (1995). One year of preschool intervention or two: Does it matter? Early Childhood Research Quarterly, 10, 1–31.

[bibr28-0193841X20976527] Reynolds A. J. (2000). Success in early intervention: The Chicago Child-Parent Centers and youth through age 15. Lincoln: University of Nebraska Press.

[bibr29-0193841X20976527] Reynolds A. J. (2008). Strong design and comprehensive analysis in the Child-Parent Center Study. Archives of Pediatrics & Adolescent Medicine, 162(11), 1099–1101. 18981363 10.1001/archpedi.162.11.1099

[bibr55-0193841X20976527] Reynolds A. J. Mehana M. Temple J. A. (1995). Does preschool intervention affect children's perceived competence? Journal of Applied Developmental Psychology, 16(2), 211–230. 10.1016/0193-3973(95)90033-0

[bibr56-0193841X20976527] Reynolds A. J. Mondi C. F. (2016). Child-Parent Centers. In Couchenour D. Chrisman J. K. (Eds.), SAGE Encyclopedia of Contemporary Early Childhood Education. SAGE Publishing.

[bibr31-0193841X20976527] Reynolds A. J. Ou S. (2009). Technical appendix: Paths of effects from preschool to adult-well-being: A confirmatory analysis of the Child-Parent Center Program. Chicago Longitudinal Study. 10.1111/j.1467-8624.2010.01562.x PMC3793348 21410923

[bibr32-0193841X20976527] Reynolds A. J. Ou S. (2011b). Paths of effects from preschool to adult well-being: A confirmatory analysis of the Child-Parent Center Program. Child Development, 82(2), 555–582. 10.1111/j.1467-8624.2010.01562.x 21410923 PMC3793348

[bibr57-0193841X20976527] Reynolds A. J. Ou S. Temple J. A. (2018). A multicomponent, preschool to third grade preventive intervention and educational attainment at 35 years of age. JAMA Pediatrics, 172(3), 247–256. 10.1001/jamapediatrics.2017.4673 29379955 PMC5885840

[bibr33-0193841X20976527] Reynolds A. J. Robertson D. L. (2003). School-based early intervention and later child maltreatment in the Chicago Longitudinal Study. Child Development, 74, 3–26. 12625433 10.1111/1467-8624.00518

[bibr34-0193841X20976527] Reynolds A. J. Temple J. A. (1995). Quasi-experimental estimates of the effects of a preschool intervention: Psychometric and econometric comparisons. Evaluation Review, 19(4), 347–373.

[bibr36-0193841X20976527] Reynolds A. J. Temple J. A. Ou S. (2003). School-based early intervention and child well-being in the Chicago Longitudinal Study. Child Welfare, 82(5), 633–656. 14524429

[bibr37-0193841X20976527] Reynolds A. J. Temple J. A. Ou S. Arteaga I. A. White B. A. B. (2011b). School-based early childhood education and age 28 well-being: Effects by timing, dosage, and subgroups. Science, 333(6040), 360–364. 10.1126/science.1203618 21659565 PMC3774305

[bibr38-0193841X20976527] Reynolds A. J. Temple J. A. Ou S. Robertson D. L. Mersky J. P. Topitzes J. W. Niles M. D. (2007). Effects of a school-based, early childhood intervention on adult health and well-being: A 19-year follow up of low-income families. Archives of Pediatrics & Adolescent Medicine, 161(8), 730–739. 10.1001/archpedi.161.8.730 17679653

[bibr39-0193841X20976527] Reynolds A. J. Temple J. A. Robertson D. L. Mann E. A. (2001). Long-term effects of an early childhood intervention on educational achievement and juvenile arrest: A 15-year follow-up of low-income children in public schools. Journal of the American Medical Association, 285, 2339–2346. 11343481 10.1001/jama.285.18.2339

[bibr40-0193841X20976527] Reynolds A. J. Temple J. A. White B. A. Ou S. Robertson D. L. (2011a). Age-26 cost-benefit analysis of the Child-Parent Center Early Education Program. Child Development, 82, 782–804. 10.1111/j.1467-8624.2010.01563.x PMC3817956 21291448

[bibr41-0193841X20976527] Rosenbaum P. R. Rubin D. B. (1983). The central role of the propensity score in observational studies for causal effects. Biometrika, 70(1), 41–55.

[bibr43-0193841X20976527] Seaman S. R. White I. R. (2013). Review of inverse probability weighting for dealing with missing data. Statistical Methods in Medical Research, 22(3), 278–295. 10.1177/0962280210395740 21220355

[bibr44-0193841X20976527] Shrout P. E. Yager T. J. (1989). Reliability and validity of screening scales: Effect of reducing scale length. Journal of Clinical Epidemiology, 42(1), 69–78. 10.1016/0895-4356(89)90027-9 2913189

[bibr58-0193841X20976527] Temple J. A. Reynolds A. J. Miedel W. T. (2000). Can early intervention prevent high school dropout?: Evidence from the Chicago child-parent centers. Urban Education, 35(1), 31–56.

[bibr45-0193841X20976527] Wittayanukorn S. Qian J. Hanson R. A. (2014). Prevalence of depressive symptoms and predictors of treatment among U. S. adults from 2005 to 2010. General Hospital Psychiatry, 36, 330–336. 24462337 10.1016/j.genhosppsych.2013.12.009

[bibr46-0193841X20976527] World Health Organization. (2017). Depression and other common mental disorders: Global health estimates. https://www.who.int/mental_health/management/depression/prevalence_global_health_estimates/en/

