# Supplementary material for: Early Childhood Education and Adult Depression: An Attrition Reanalysis With Inverse Propensity Score Weighting
Source: Eval Rev. 2020 Dec 14;44(5-6):379–409. doi: 10.1177/0193841X20976527 (PMC8127666; doi:10.1177/0193841X20976527)
Supplement: Supplemental Material, sj-docx-1-erx-10.1177_0193841X20976527 - Early Childhood Education and Adult Depression: An Attrition Reanalysis With Inverse Propensity Score Weighting [file sj-docx-1-erx-10.1177_0193841X20976527.docx]

**Appendix A**

**Covariates in the Outcome Model**

*Sex.* Information about sex was drawn from administrative school records. Females were assigned a dichotomous code of “1”; males were assigned a code of “0”.

*Race/ethnicity.* Information about race/ethnicity was drawn from administrative school records. African American participants were assigned a dichotomous code of “1”; all others were assigned a code of “0”.

*Low birth weight.* Information about birth weight was drawn from participants’ birth certificates. Participants who were of low birthweight (<2500 grams) were assigned a dichotomous code of “1”; all others were assigned a code of “0”.

*CPC preschool participation.* Information about CPC preschool participation was drawn from administrative school records. Participants who attended CPC preschool were assigned a dichotomous code of “1”; participants who did not participate in the preschool program were assigned a code of “0”.

*CPC school-age participation.* Information about participation in the school-age CPC program was drawn from administrative school records. Participants who attended the school-age CPC program between Grades 1-3 were assigned a dichotomous code of “1”; all others were assigned a code of “0”.

*Family risk index (ages zero to three).* An index of participants’ socioeconomic risk status between ages zero to three years was computed by summing eight risk factors that were dichotomously coded for presence (1) or absence (0): (a) mother was under age 18 at the participant’s birth; (b) mother was not a high school graduate; (c) mother was unemployed or employed part-time; (d) participant lived in a single parent household; (e) participant lived in a household of four or more children; (f) participant lived in a school attendance area where at least 60% of households were impoverished; (g) participant’s family income was below 185% of the federal poverty level; and (h) participant was eligible for free lunch. Information was drawn from family surveys and school records.

*Family conflict (ages zero to five).* Participants indicated whether they had experienced “frequent family conflict” between zero and five years of age on the age 22-24 survey. Participants who endorsed frequent family conflict were assigned a dichotomous code of “1”; all others were assigned a code of “0”.

*Substance abuse of a parent (ages zero to five).* Participants indicated whether they had experienced “problem of substance abuse of parent” between zero and five years of age on the age 22-24 survey. Participants who endorsed parental substance abuse were assigned a dichotomous code of “1”; all others were assigned a code of “0”.

*Family financial problems (ages zero to five).* Participants indicated whether they had experienced “family financial problems” between zero and five years of age on the age 22-24 survey. Participants who endorsed family financial problems were assigned a dichotomous code of “1”; all others were assigned a code of “0”.

*Survey completion date.* Participants who completed the survey in 2002 were assigned a code of “1”; participants who completed the survey in 2003 or later were assigned a code of “0”.

*Survey completion by mail.* Participants who completed a hard copy of the survey and mailed it back to the research laboratory were assigned a code of “1”; all others were assigned a code of “0”.

*Survey completion in person.* Participants who completed the survey in person with a research staff member were assigned a code of “1”; all others were assigned a code of “0”.

**Appendix B**

Table B1

*Balance check: Regression model predicting depression symptoms at age 22-24, with age 0-5 composite risk index, including year and mode of survey completion, with IPW for age 22-24 attrition*

|  | Standardized difference |
| --- | --- |
| Female | -0.03 |
| African American | -0.02 |
| Low birth weight | -0.02 |
| CPC Preschool | 0.03 |
| CPC School-Age Participation | 0.03 |
| Kindergarten word-identification score | 0.00 |
| Composite Kindergarten school readiness score | -0.03 |
| Substantiated maltreatment (age 0-3) | -0.01 |
| Mother was not a high school graduate (by participant age 3) | -0.01 |
| Eligible for free lunch (age 0-3) | 0.03 |
| Mother was under 18 at participant’s birth | -0.07 |
| Lived in a household of four or more children (age 0-3) | 0.04 |
| Family income below 185% of the federal poverty level (age 0-3) | 0.02 |
| Mother was unemployed or employed part-time (age 0-3) | 0.04 |
| Lived in a single parent household (between age 0-3) | 0.02 |
| Lived in a school attendance area where at least 60% of households were impoverished (age 0-3) | 0.00 |
| Information is missing for at least one age 0-3 risk indicator | 0.03 |

| Table B1 (continued) |  |
| --- | --- |
|  | Standardized difference |
| Frequent family conflict (age 0-5) | 0.03 |
| Family financial problems (age 0-5) | -0.02 |
| Parental substance abuse (age 0-5) | 0.01 |
| Active in Chicago Public Schools for 6+ years between Kindergarten and 8^th^ grade | 0.02 |
| Mother participated in at least two years of postsecondary education (by participant age 17) | -0.04 |
| Number of school moves between Kindergarten and grade 4 | 0.02 |
| % of individuals living 1 year within the participant’s housing unit (by participant age 4) | 0.06 |
| % of individuals living 1-5 years within the participant’s housing unit (by participant age 4) | -0.02 |
| % of individuals living 5-10 years within the participant’s housing unit (by participant age 4) | -0.03 |
| % of individuals living 10-20 years within the participant’s housing unit (by participant age 4) | -0.03 |
| % of self-employed individuals ages 16+ within the participant’s census tract (by participant age 4) | 0.01 |
| % of female-headed black households within the participant’s census tract (by participant age 4) | 0.00 |

| Table B1 Continued |  |
| --- | --- |
|  | Standardized difference |
| Magnet school attendance (grades 4-8) | -0.01 |
| 8^th^ grade reading score | 0.02 |
| Juvenile arrest | -0.01 |
| Social Security number identified by 2007 | -0.02 |

**p*<0.05; ***p*<0.01; ****p*<0.001

Table B2

*Regression model predicting depression symptoms at age 22-24, with age 0-5 composite risk index, not including year or mode of survey completion, with IPW for age 22-24 attrition*

|  | CPC Preschool Group | | Comparison Group | |
| --- | --- | --- | --- | --- |
|  | dy/dx | 95% CI | dy/dx | 95% CI |
| CPC School-Age Participation | -0.03 | -0.08 – 0.03 | 0.12* | 0.02 – 0.21 |
| Black | 0.10*** | 0.05 – 0.15 | 0.12* | 0.03 – 0.22 |
| Female | 0.02 | -0.03 – 0.06 | -0.09 | -0.16 – -0.01 |
| Low Birth Weight | -0.05 | -0.10 – 0.01 | 0.00 | -0.11 – 0.12 |
| Family Risk (ages 0-3) | 0.02*** | 0.01 - 0.04 | 0.01 | -0.01 – 0.03 |
| Family Conflict (ages 0-5) | 0.10 | -0.02 – 0.22 | 0.14 | -0.10 – 0.38 |
| Parental Substance Abuse (ages 0-5) | -0.06 | -0.13 – 0.00 | 0.12 | -0.19 – 0.42 |
| Family Financial Problems (ages 0-5) | 0.21** | 0.08 – 0.33 | -0.02 | -0.18 – 0.15 |
| Mean Point Difference in % of 1+ Depression Symptoms (age 22-24) | 7.4 points [CI: 6.7-8.0 points) | | | |

**p*<0.05; ***p*<0.01; ****p*<0.001

CI = Confidence Interval

Table B3

*Balance Check: Regression model predicting depression symptoms at age 22-24, with age 0-5 composite risk index, not including year or mode of survey completion, with IPW for age 22-24 attrition*

|  | Standardized difference |
| --- | --- |
| Female | -0.03 |
| African American | -0.02 |
| Low birth weight | -0.02 |
| CPC Preschool | 0.03 |
| CPC School-Age Participation | 0.03 |
| Kindergarten word-identification score | 0.00 |
| Composite Kindergarten school readiness score | -0.03 |
| Substantiated maltreatment (age 0-3) | -0.01 |
| Mother was not a high school graduate (by participant age 3) | -0.01 |
| Eligible for free lunch (age 0-3) | 0.03 |
| Mother was under 18 at participant’s birth | -0.07 |
| Lived in a household of four or more children (age 0-3) | 0.04 |
| Family income below 185% of the federal poverty level (age 0-3) | 0.02 |
| Mother was unemployed or employed part-time (age 0-3) | 0.04 |
| Lived in a single parent household (between age 0-3) | 0.02 |
| Lived in a school attendance area where at least 60% of households were impoverished (age 0-3) | 0.00 |
| Information is missing for at least one age 0-3 risk indicator | 0.03 |

| Table B3 (continued) |  |
| --- | --- |
|  | Standardized difference |
| Frequent family conflict (age 0-5) | 0.03 |
| Family financial problems (age 0-5) | -0.02 |
| Parental substance abuse (age 0-5) | 0.01 |
| Active in Chicago Public Schools for 6+ years between Kindergarten and 8^th^ grade | 0.02 |
| Mother participated in at least two years of postsecondary education (by participant age 17) | -0.04 |
| Number of school moves between Kindergarten and grade 4 | 0.02 |
| % of individuals living 1 year within the participant’s housing unit (by participant age 4) | 0.06 |
| % of individuals living 1-5 years within the participant’s housing unit (by participant age 4) | -0.02 |
| % of individuals living 5-10 years within the participant’s housing unit (by participant age 4) | -0.03 |
| % of individuals living 10-20 years within the participant’s housing unit (by participant age 4) | -0.03 |
| % of self-employed individuals ages 16+ within the participant’s census tract (by participant age 4) | 0.01 |
| % of female-headed black households within the participant’s census tract (by participant age 4) | 0.00 |

| Table B3 (continued) |  |
| --- | --- |
|  | Standardized difference |
| Magnet school attendance (grades 4-8) | -0.01 |
| 8^th^ grade reading score | 0.02 |
| Juvenile arrest | -0.01 |
| Social Security number identified by 2007 | -0.02 |

**p*<0.05; ***p*<0.01; ****p*<0.001

Table B4

*Regression model predicting depression symptoms at age 22-24, with age 0-5 individual risk indicators, including year and mode of survey completion, with IPW for age 22-24 attrition*

|  | CPC Preschool Group | | Comparison Group | |
| --- | --- | --- | --- | --- |
|  | dy/dx | 95% CI | dy/dx | 95% CI |
| CPC School-Age Participation | -0.03 | -0.08 – 0.02 | 0.11* | 0.01 – 0.20 |
| Black | 0.09** | 0.03 – 0.14 | 0.13*** | 0.06 – 0.21 |
| Female | 0.16 | -0.03 – 0.06 | -0.06 | -0.13 – 0.02 |
| Low Birth Weight | -0.04 | -0.09 – 0.02 | 0.03 | -0.09 – 0.15 |
| Mother was not a high school graduate (by participant age 3) | 0.02 | -0.03 – 0.06 | 0.05 | -0.03 – 0.14 |
| Eligible for free lunch (age 0-3) | 0.00 | -0.08 – 0.08 | -0.05 | -0.16 – 0.07 |
| Mother was under 18 at participant’s birth | -0.05 | 0.00 – 0.16 | 0.02 | -0.08 – 0.13 |
| Lived in a household of four or more children (age 0-3) | 0.05 | -0.03 – 0.12 | -0.02 | -0.11 – 0.07 |
| Family income below 185% of the federal poverty level (age 0-3) | 0.04 | -0.03 – 0.10 | -0.09 | -0.10 – 0.07 |
| Mother was unemployed or employed part-time (age 0-3) | 0.03 | -0.03 – 0.09 | 0.12* | 0.01 – 0.22 |
| Table B4 (continued) |  |  |  |  |
|  | CPC Preschool Group | | Comparison Group | |
|  | dy/dx | 95% CI | dy/dx | 95% CI |
| Lived in a single parent household (between age 0-3) | 0.07** | 0.03 – 0.12 | -0.03 | -0.14 – 0.07 |
| Lived in a school attendance area where at least 60% of households were impoverished (age 0-3) | 0.00 | -0.05 – 0.06 | -0.01 | -0.10 – 0.07 |
| Family Conflict (ages 0-5) | 0.10 | -0.03 – 0.22 | 0.08 | -0.12 – 0.27 |
| Parental Substance Abuse (ages 0-5) | -0.06 | -0.12 – -0.01 | 0.10 | -0.21 – 0.40 |
| Family Financial Problems (ages 0-5) | 0.19** | 0.06 – 0.31 | 0.00 | -0.18 – 0.17 |
| Survey Completed in 2002 (age 22-24) | -0.01 | -0.06 – 0.04 | -0.07 | -0.16 – 0.02 |
| Survey Returned via Mail (age 22-24) | 0.10 | -0.01 – 0.21 | 0.14 | -0.03 – 0.30 |
| Survey Completed In-Person (age 22-24) | -0.01 | -0.08 – 0.06 | 0.01 | -0.11 – 0.12 |
| Mean Point Difference in % of 1+ Depression Symptoms (age 22-24) | 7.1 points [CI: 5.4-9.7 points) | | | |

**p*<0.05; ***p*<0.01; ****p*<0.001

CI = Confidence Interval

Table B5

*Regression model predicting depression symptoms at age 22-24, with age 0-5 composite risk index, including year and mode of survey completion, with IPW for treatment selection*

|  | CPC Preschool Group | | Comparison Group | |
| --- | --- | --- | --- | --- |
|  | dy/dx | 95% CI | dy/dx | 95% CI |
| CPC School-Age Participation | -0.04 | -0.09 – 0.02 | 0.11* | 0.02 – 0.20 |
| Black | 0.08* | 0.01 – 0.15 | 0.11* | 0.02 – 0.20 |
| Female | 0.02 | -0.03 -0.07 | -0.04 | -0.12 – 0.04 |
| Low Birth Weight | -0.03 | -0.09 – 0.03 | 0.00 | -0.11 – 0.11 |
| Family Risk (ages 0-3) | 0.02** | 0.01 – 0.04 | 0.02 | 0.00 – 0.04 |
| Family Conflict (ages 0-5) | 0.11 | -0.03 – 0.24 | 0.16 | -0.08 – 0.40 |
| Parental Substance Abuse (ages 0-5) | -0.07* | -012 - -0.01 | 0.05 | -0.26 – 0.36 |
| Family Financial Problems (ages 0-5) | 0.19** | 0.06 – 0.32 | 0.00 | -0.17 – 0.18 |
| Survey Completed in 2002 (age 22-24) | -0.01 | -0.06 – 0.05 | -0.08 | -0.18 – 0.01 |
| Survey Returned via Mail (age 22-24) | 0.11 | 0.00 – 0.22 | 0.06 | -0.08 – 0.21 |

| Table B5 (continued) |  |  |  |  |
| --- | --- | --- | --- | --- |
|  | CPC Preschool Group | | Comparison Group | |
|  | dy/dx | 95% CI | dy/dx | 95% CI |
| Survey Completed In-Person (age 22-24) | -0.01 | -0.08 – 0.07 | 0.00 | -0.12 – 0.12 |
| Mean Point Difference in % of 1+ Depression Symptoms (age 22-24) | 7.3 points [CI: 1.9 – 12.9 points) | | | |

**p*<0.05; ***p*<0.01; ****p*<0.001

CI = Confidence Interval

Table B6

*Regression model predicting depression symptoms at age 22-24, with age 0-5 composite risk index, not including year and mode of survey completion, with IPW for treatment selection and age 22-24 attrition*

|  | CPC Preschool Group | | Comparison Group | |
| --- | --- | --- | --- | --- |
|  | dy/dx | 95% CI | dy/dx | 95% CI |
| CPC School-Age Participation | -0.03 | -0.08 – 0.03 | 0.13** | 0.04 – 0.23 |
| Black | 0.09** | 0.04 – 0.15 | 0.12** | 0.03 – 0.21 |
| Female | 0.02 | -0.03 – 0.07 | -0.05 | -0.13 – 0.04 |
| Low Birth Weight | -0.04 | -0.10 – 0.02 | 0.00 | -0.12 – 0.12 |
| Family Risk (ages 0-3) | 0.02** | 0.01 – 0.04 | 0.02 | -0.01 – 0.04 |
| Family Conflict (ages 0-5) | 0.11 | -0.02 – 0.25 | 0.15 | -0.10 – 0.40 |
| Parental Substance Abuse (ages 0-5) | -0.07** | -0.13 – -0.02 | 0.09 | -0.24 – 0.42 |
| Family Financial Problems (ages 0-5) | 0.19** | 0.06 – 0.32 | -0.03 | -0.19 – 0.14 |
| Survey Completed in 2002 (age 22-24) | -0.01 | -0.06 – 0.05 | -0.08 | -0.18 – 0.02 |
| Survey Returned via Mail (age 22-24) | 0.10 | -0.09 – 0.21 | 0.08 | -0.08 – 0.24 |

| Table B6 (continued) |  |  |  |  |
| --- | --- | --- | --- | --- |
|  | CPC Preschool Group | | Comparison Group | |
|  |  |  |  |  |
| Survey Completed In-Person (age 22-24) | -0.01 | -0.09 – 0.06 | 0.00 | -0.13 – 0.12 |
| Mean Point Difference in % of 1+ Depression Symptoms (age 22-24) | 7.9 points [CI: 4.9 – 10.7 points) | | | |

*p<0.05; **p<0.01; ***p<0.001

CI = Confidence Interval

Table B7

*Monte Carlo Simulation Results for 10,000 Iterations: Child-Parent Center (CPC) Preschool and Comparison Groups on Depressive Symptoms at Ages 22-24*

| Metric | Mean | SD | Min. | Max. | Pct. of tests  exceeding 95% CI,  2007 study | Change from 2007 study |
| --- | --- | --- | --- | --- | --- | --- |
| Difference in percentage points | -7.87 | 1.48 | -2.36 | -13.68 | 15.22 | -3.27 |
| Effect size in SD | -0.36 | 0.07 | -0.199 | -0.528 | n/a | -0.16 |
| Percent change over comparison | -42.77 | 8.04 | -12.83 | -74.34 | n/a | -16.4 |

*Note*. Monte Carlo analysis tested the range of point estimates (main effects) and the percentage of iterations that the estimate in the reanalysis exceeded the confidence interval of the program effect from the original paper (Reynolds et al., 2007). The mean standard error across the iterations (95% CI) was 0.015 (-7.84, -7.90). Alternative metrics are provided as are change values relative to the 2007 study. The percent change over the comparison group is based on the unadjusted mean (prevalence rate) for this group (18.4%; .0787/.184 = 42.77%). Effects sizes were calculated using the probit transformation of percentages (10.53% [program] vs. 18.4% [comparison]). ESs for unadjusted, 2007 study, and reanalysis are -0.21, -0.20, and -0.32, respectively.
